# Thermoresponsive Interplay of Water Insoluble Poly(2-alkyl-2-oxazoline)s Composition and Supramolecular Host–Guest Interactions

**DOI:** 10.3390/ijms16047428

**Published:** 2015-04-02

**Authors:** Victor R. de la Rosa, Werner M. Nau, Richard Hoogenboom

**Affiliations:** 1 Supramolecular Chemistry Group, Department of Organic and Macromolecular Chemistry, Ghent University, Krijgslaan 281 S4, 9000 Ghent, Belgium; E-Mail: victor.retamerodelarosa@ugent.be; 2 Department of Life Sciences and Chemistry, Jacobs University Bremen, Campus Ring 1, D 28759 Bremen, Germany; E-Mail: w.nau@jacobs-university.de

**Keywords:** Lower Critical Solution Temperature (LCST), temperature responsive polymers, poly(2-alkyl-2-oxazoline)s, cyclodextrins, cucurbit[7]uril, supramolecular interactions, nanoparticles

## Abstract

A series of water insoluble poly[(2-ethyl-2-oxazoline)-*ran*-(2-nonyl-2-oxazoline)] amphiphilic copolymers was synthesized and their solubility properties in the presence of different supramolecular host molecules were investigated. The resulting polymer-cavitand assemblies exhibited a thermoresponsive behavior that could be modulated by variation of the copolymer composition and length. Interestingly, the large number of hydrophobic nonyl units across the polymer chain induced the formation of kinetically-trapped nanoparticles in solution. These nanoparticles further agglomerate into larger aggregates at a temperature that is dependent on the polymer composition and the cavitand type and concentration. The present research expands the understanding on the supramolecular interactions between water insoluble copolymers and supramolecular host molecules.

## 1. Introduction

Thermoresponsive polymers, able to respond to changes in the environmental temperature, constitute a fascinating polymer class that has resulted in a myriad of applications in fields as diverse as construction [1,2], water management [3], separation sciences [4,5], or shape memory materials [6]. In solution, thermoresponsive polymers result in the so called smart fluids or smart soluble materials, which exhibit parallels with natural responsive systems [7]. In this context, thermoresponsive polymers have become an invaluable tool to develop new applications in biomedicine [8], especially in drug and gene delivery [9], tissue engineering [10] and as sensors [11,12].

In contrast to their natural counterparts that usually exhibit an upper critical solution temperature (UCST), most synthetic thermoresponsive polymers exhibit a lower critical solution temperature (LCST) behavior, which arises from their negative entropy of solvation. This involves a sharp polymer phase separation upon heating the solution above a specific temperature, a feature that has been successfully exploited in the development of soluble temperature sensors that operate at the nanoscale [12,13,14,15,16]. The polymer transition temperature depends upon polymer molecular mass, composition and concentration and is regarded as cloud point temperature (T_CP_). Controlling the hydrophobicity of the polymer thus allows the tuning of the T_CP_ to tailor it to the required application, as has been extensively investigated by varying the polymer composition [17], e.g., in poly(oligo ethylene glycol (meth)acrylate)s [18], poly(2-hydroxypropyl acrylate)s [19], and poly(2-oxazoline)s [20,21,22,23,24,25]. In particular, copolymerization of hydrophobic 2-nonyl-2-oxazoline (NonOx) and hydrophilic 2-ethyl-2-oxazoline (EtOx) yields random copolymers whose thermal responsiveness can be easily tuned by the ratio of both monomers [26,27]. In addition, the polymer hydrophilic-hydrophobic balance can be controlled by specific supramolecular interactions with amphiphilic host molecules, as has been found in end-group modified poly(2-alkyl-2-oxazoline)s (PAOx) and in other polymer platforms [28,29,30,31,32]. Cavitands can form complexes with matching guest units along the polymer chain offering control on the ensemble’s transition temperature [33] and morphology [34,35,36,37,38,39] in solution, as has also been investigated for crown-ether decorated polymers [40,41].

Recently, we have studied the influence of a range of different supramolecular hosts on the solubility properties of a thermoresponsive PEtOx-*ran*-PNonOx random copolymer containing 12 mol % nonyl side chains, wherein the nonyl chains act as guest units for the cavitands [42]. The cavitands tested were selected among well-known hosts for aliphatic chains: cucurbit[7]uril (CB7), alpha-cyclodextrin (αCD), and hydroxypropylated alpha- and beta-cyclodextrins (HPαCD and HPβCD, respectively). Besides obtaining an unprecedentedly broad tunability of the T_CP_ of *ca*. 30 K with stoichiometric amounts of cavitand, the extent of the transition was found to be directly correlated to the polymer-cavitand host–guest binding affinity. Indeed, the power of each cavitand to increase the T_CP_ of the polymer-cavitand ensemble followed the order of nonyl-cavitand binding affinity, *i.e.*, CB7 (K_a_ ≈ 2200 M^−1^) >> αCD (K_a_ ≈ 440 M^−1^) > HPαCD (K_a_ ≈ 220 M^−1^) > HPβCD (K_a_ ≈ 120 M^−1^) [42]. In a second report, we demonstrated that fully water-insoluble PEtOx-*ran*-PNonOx copolymers containing 33 mol % NonOx assemble into defined nanoparticles in presence of excess cavitands. Interestingly, these nanoparticles either reversibly or irreversibly agglomerated upon heating enabling their use as temperature sensors with rewritable or permanent memory functions, respectively, depending on the utilized cavitands [43].

**Figure 1:**
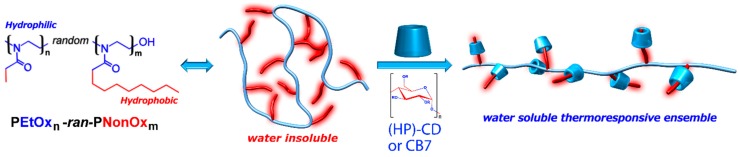
A series of amphiphilic PEtOx-*ran*-PNonOx random copolymers was synthesized and their solubility properties were studied in the presence of a range of different supramolecular host molecules. The picture describes the supramolecular complexation of a PEtOx-*ran*-PNonOx random copolymer with cavitands resulting in the formation of thermoresponsive supramolecular complexes.

In the present study, we have investigated the effect of increasing the polymer length and nonyl side chains content on the thermoresponsive behavior of the polymer-cavitand ensembles in further detail. All the here reported PEtOx-*ran*-PNonOx copolymers were insoluble in water at near 0 °C. The copolymers could however be brought in solution in the presence of the cavitands, leading to thermoresponsive polymer-cavitand ensembles (see Figure 1).

Interestingly, increasing the number of nonyl guest units, and consequently the strength of the polymer-polymer interactions, led to a dependence of the T_CP_ on the cavitand hydrophilicity, and not only on the strength of the nonyl-cavitand host–guest interactions. Hereby we present a detailed study on the differential interplay between supramolecular hosts and a range of water insoluble PEtOx-*ran*-PNonOx copolymers, aiming to expand the understanding on the supramolecular interactions of host molecules with water insoluble polymers.

## 2. Results and Discussion

The soluble copolymer that was studied in our previous report [42] contained 12% nonyl side chains, and a composition of PEtOx_90_-*ran*-PNonOx_12_ (nearly 100 repeating units). This copolymer exhibited a T_CP_ of *ca.* 10 °C. In the present study, we aimed to analyze the differential interaction of supramolecular hosts with highly hydrophobic PEtOx-*ran*-PNonOx random copolymers, investigating the effect of polymer composition and length. To this end, a series of PEtOx-*ran*-PNonOx random copolymers with increasing NonOx content and varying degree of polymerization was synthesized.

### 2.1. Poly(2-ethyl-2-oxazoline)-ran-poly(2-nonyl-2-oxazoline) Synthesis

**Figure 2:**
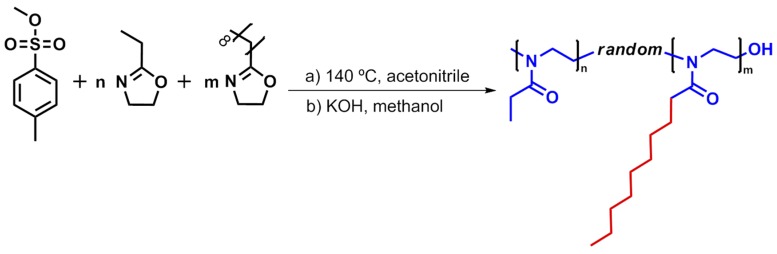
Schematic representation of the living cationic ring-opening copolymerization of 2-ethyl-2-oxazoline (EtOx) and 2-nonyl-2-oxazoline (NonOx) initiated by methyl tosylate resulting in PEtOx-*ran*-PNonOx random copolymers.

The copolymerizations were performed following a previously reported protocol [26] by preparing solutions of EtOx and NonOx with the desired stoichiometry in dry acetonitrile, and a total monomer concentration of 4 M. The initiator used was methyl tosylate (MeOTs) [44] and the monomer to initiator ratio was selected to obtain copolymers with the desired length (details are given in the Experimental Section). All polymerizations were performed to up to full conversion in capped vials in a microwave synthesizer at 140 °C and terminated with methanolic KOH, resulting in hydroxyl-terminated polymers (see Figure 2).

**Figure 3:**
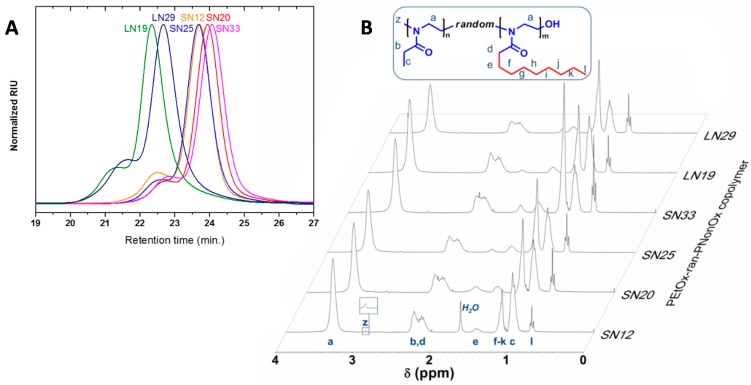
(**A**) Size exclusion chromatography (SEC) data of the synthesized PEtOx*_n_*-*ran*-PNonOx*_m_* random copolymers. Albeit a high molecular weight distribution is observed as shoulder in all cases, ascribed to chain coupling, well-defined polymers with dispersities around 1.1 were obtained. SEC eluent: *N*,*N*-dimethylacetamide containing 50 mM LiCl; (**B**) ^1^H-NMR spectra of the investigated PEtOx-*ran*-PNonOx random copolymers in CDCl_3_. Legend: S = Short polymer, DP ≈ 100, L = Long polymer DP ≈ 200. N = % Nonyl side chains in number.

Interestingly, the EtOx-NonOx statistical copolymerization results in ideal random copolymer structures, since the reactivity ratios of both monomers are equal to unity, as has been previously reported [26,45]. The obtained P[(EtOx)*_n_*-*ran*-(NonOx)*_m_*] copolymer compositions were determined by ^1^H-NMR spectroscopy (see Figure 3 and Table 1).

**Table 1:** Composition and size exclusion chromatography (SEC) data of the synthesized PEtOx*_n_*-*ran*-PNonOx*_m_* random copolymers. Polymer composition was calculated by ^1^H-NMR analysis. SEC eluent: *N*,*N*-dimethylacetamide containing 50 mM LiCl. Calibrated against PMMA standards. Polymer ID legend: S = Short polymer, DP ≈ 100, L = Long polymer DP ≈ 200.

ID	Polymer Composition	DP	% NonOx (Number)	SEC	
				M_n_	Đ
SN12	PEtOx_90_-*ran*-PNonOx_12_	102	12	18,000	1.08
SN20	PEtOx_84_-*ran*-PNonOx_21_	105	20	17,500	1.08
SN25	PEtOx_75_-*ran*-PNonOx_25_	100	25	16,500	1.07
SN33	PEtOx_62_-*ran*-PNonOx_29_	91	33	15,000	1.09
LN19	PEtOx_162_-*ran*-PNonOx_38_	200	19	29,700	1.11
LN29	PEtOx_140_-*ran*-PNonOx_57_	197	29	28,000	1.13

All the synthesized copolymers exhibited dispersities below 1.15, as determined by size exclusion chromatography, indicating good control over the polymerizations (see Figure 3). Nevertheless, as commonly observed, a high molecular weight shoulder was present in all copolymers, presumably due to chain-transfer reactions [46]. The observed differences in retention time are not only ascribed to different number average molecular weight, but also to the different hydrophobicity of the copolymers. As the content of NonOx in the copolymer increases, so does its hydrophobicity, resulting in a less efficient solvation by the polar *N*,*N*-dimethylacetamide eluent, producing a smaller hydrodynamic volume. Consequently, prolonged retention times are observed for the most hydrophobic copolymers. This effect is especially noticeable for the LN19 and LN29 copolymers, both with a degree of polymerization (DP) close to 200 repeating units; albeit LN29 has a higher molecular weight, its retention time is higher than that of LN19, indicating a distinctly larger hydrophobicity. Later on, these preliminary observations will translate into marked differences in the copolymer solubility properties in the presence of cavitands.

### 2.2. Influence of Nonyl Side Chain Content: Poly(2-ethyl-2-oxazoline)-ran-poly(2-nonyl-2-oxazoline) Containing 20% Nonyl Chains

Initially, we investigated a PEtOx_84_-*ran*-PNonOx_21_ copolymer containing 20% nonyl chains in number, therefore roughly doubling the NonOx content of the previously reported PEtOx_90_-*ran*-PNonOx_12_ copolymer, while maintaining its length at *ca.* 100 repeating units. The additional NonOx content made this copolymer insoluble in water, and therefore the addition of host molecules to the solution, followed by freezing and thawing under sonication in an ice-bath, was necessary to bring the copolymer in solution. This protocol seemed to favor the formation of inclusion complexes with the nonyl chains affording the solubilization of the copolymer.

**Figure 4:**
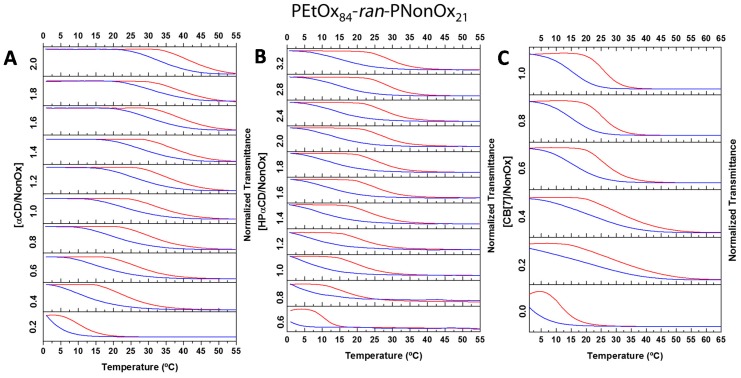
Turbidimetry studies of P[(EtOx)_84_-*ran*-(NonOx)_21_] with increasing cavitand/NonOx molar ratios. 5 mg·mL^−1^ solutions of copolymer were titrated with αCD (**A**) and HPαCD (**B**), whereas a 2 mg·mL^−1^ solution of copolymer was titrated with CB7 (**C**). The heating and cooling ramps are represented by the top (red) and bottom (blue) curves, respectively. Rate: 1 K·min^−1^, λ = 700 nm.

A 5 mg·mL^−1^ solution of the PEtOx_84_-*ran*-PNonOx_21_ random copolymer was prepared and titrated with different cavitands, while monitoring the evolution of the copolymer T_CP_. The cavitands that previously exhibited a stronger binding constant with nonyl chains were selected to perform these studies, namely CB7, αCD, and HPαCD. Interestingly, the hydrophilicity order of these cavitands is the inverse of their binding affinity to nonyl chains, *i.e.*, HPαCD >> αCD > CB7, with water solubilities that are, respectively, >600, 145 and 39 mg·mL^−1^ [47,48]. To be able to titrate CB7, due to its relatively low solubility, a 2 mg·mL^−1^ solution of the copolymer was used. As previously found for the PEtOx-*ran*-PNonOx containing 12% NonOx, the temperature-triggered phase transition of all solutions could be tuned by the addition of the cavitands (see Figure 4).

**Figure 5:**
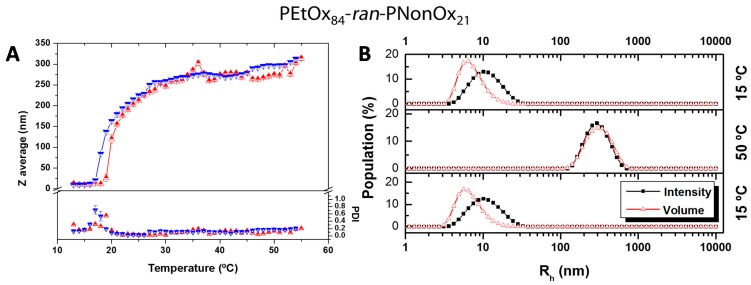
Temperature-dependent Dynamic Light Scattering (DLS) data for a 5 mg·mL^−1^ solution of P[(EtOx)_84_-*ran*-(NonOx)_21_] and 1.0 equivalent of αCD (relative to nonyl side chains). (**A**) Z average and PDI evolution with temperature. >heating; Icooling. Random coil copolymer-cavitand ensembles are formed at low temperature and are stable up to ≈20 °C (transparent solution); above this temperature, the copolymer-cavitand ensembles re-arrange into well-defined nanoparticles (279 nm, PDI = 0.090 at 50 °C). Four measurements are averaged at every temperature. Approximate heating/cooling rate: ≈0.01 K·min^−1^; (**B**) Particle size distribution of the sample at 15, 50 °C and back to 15 °C. At 15 °C individual copolymer-cavitand ensembles are observed. Heating beyond 20 °C leads to the formation of well-defined nanoparticles that slowly grow with temperature (279 nm, PDI = 0.090 at 50 °C). Back to 15 °C, the host–guest complexes re-form and random coil copolymer ensembles are observed (10 nm, PDI = 0.136).

However, instead of a sharp shift in transmittance, the transition is now much more progressive. Since the solubility phase-transition is produced by the disassembly of the host–guest complexes, the progressive transition observed here indicates slow kinetics in the breakage/formation of the cavitand-nonyl host–guest complexes. This can be explained by the larger hydrophobicity of the copolymer that forms kinetically-trapped nanoparticles entrapping the cavitands and partially isolating them from the surrounding aqueous solution. This hypothesis was supported by temperature-dependent dynamic light scattering (DLS) measurements of a solution containing the copolymer and 1.0 equivalent αCD, which showed the formation of well-defined nanoparticles beyond *ca.* 20 °C. As seen in Figure 5, at low temperatures a single particle distribution of *ca.* 10 nm in size (PDI = 0.15) is observed. This is ascribed to the formation of well hydrated random-coil copolymer-cavitand ensembles, and is analogous to the behavior observed for the previously studied copolymer containing 12% NonOx. When this copolymer was heated beyond its T_CP_ in the presence of 1.0 equivalent αCD, large >1000 nm aggregates were formed, indicating de-threading of the αCD units and the collapse of the polymer chains into thermodynamically stable aggregates (data not shown). However, in the case of the PEtOx_84_-*ran*-PNonOx_21_ copolymer, upon heating beyond the T_CP_ at *ca.* 22 °C, instead of large aggregates, the formation of moderate size well-defined nanoparticles is observed (≈250 nm, PDI < 0.15). The slow heating/cooling rate applied in the DLS experiments (≈0.01 K·min^−1^) provides sufficient time for the copolymer-cavitand ensembles to change their conformation and adapt to the progressive temperature-induced breakage of nonyl-cavitand inclusion complexes. The nanoparticles slowly increased in size with temperature, possibly due to the incorporation of additional copolymer chains during heating or due to further agglomeration of the initially formed nanoparticles.

It should also be noted that the apparent hysteresis observed in the turbidimetry measurements originates from the relatively fast heating/cooling rate of 1 K·min^−1^ applied, as it is not seen in the DLS experiments, were a much lower rate was applied.

The titration with HPαCD yielded markedly different results, as 0.6 equivalents of HPαCD were necessary to solubilize the copolymer at near-zero degrees, in contrast to only 0.2 equivalents of αCD. Although HPαCD has a much larger hydrophilicity than native αCD, its lower binding affinity to nonyl chains results in a much lower ability to increase the solubility of the copolymer. On the other hand, the similar progressive decay in transmittance in the presence of HPαCD suggests the formation of kinetically-trapped structures as in the case of αCD. The low impact that increasing concentration of HPαCD has on the T_CP_ indicates that a large number of nonyl chains is shielded from the surrounding aqueous environment, further supporting the formation of kinetically trapped nanoparticles with a hydrophobic core.

**Figure 6:**
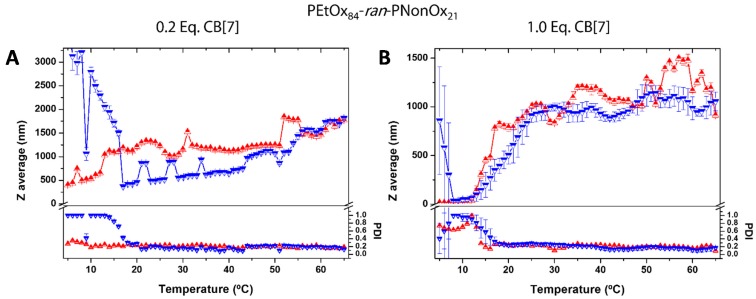
Temperature-dependent Dynamic Light Scattering (DLS) data for a 2 mg·mL^−1^ solution of P[(EtOx)_84_-*ran*-(NonOx)_21_] in the presence of 0.2 or 1.0 equivalent of CB7 (relative to nonyl side chains). Z average and PDI evolution with temperature: >heating; Icooling. (**A**) 0.2 Eq. CB7: At low temperatures, ill-defined aggregates are formed. Upon heating, macromolecular precipitates appear, that partially re-dissolve upon cooling below 20 °C, resulting in an increase in the Z_ave_; (**B**) 1.0 Eq. CB7: At low temperature, random coil ensembles (≈9 nm) coexist with aggregates of sizes from 40 to 300 nm. Beyond 20 °C, large aggregates are formed. Four measurements are averaged at every temperature. Approximate heating/cooling rate: ≈0.01 K·min^−1^.

On the other hand, CB7 produced an apparent large impact on the T_CP_ at low CB7 concentrations but, overall, did not have a large effect on the copolymer T_CP_. Temperature dependent DLS studies were performed in samples containing 0.2 and 1.0 equivalents of CB7 to investigate the reasons behind this behavior. The sample containing 0.2 equivalents of CB7 showed the absence of nanoparticle formation and the presence of large aggregates, even at low temperatures. This is manifested by the high PDI values observed below 20 °C by DLS (Figure 6). Possibly, the low hydrophilicity of the macromolecule constitutes an obstacle to disrupt the compact copolymer globules, which are stabilized by hydrophobic interactions among the nonyl side chains. Upon heating, macromolecular precipitates appear, that partially re-dissolve upon cooling below 20 °C, resulting in an apparent increase in the Z_ave_. One equivalent of the cavitand was sufficient to form random coil copolymer ensembles of ≈9 nm in size at low temperatures, but these coexisted with aggregates with a variety of sizes (40–300 nm, PDI ≈ 0.8). Heating to temperatures above 20 °C produced the collapse of the aggregates into large particles in the micrometer range. The presence of aggregates in solution, even at low temperatures, indicate the inability of CB7 to produce kinetically trapped nanoparticles, possibly due to its lower hydrophilicity compared to αCD and HPαCD.

To summarize, the increment in NonOx content in the copolymer to 20% had a strong impact on its solubility properties when compared with the previously studied copolymer with 12% NonOx. The copolymer was water insoluble and could only be brought in solution in the presence of cavitands. The increased number of nonyl groups per copolymer chain induced the formation of kinetically-trapped nanoparticles in solution when sufficiently hydrophilic cavitands, *i.e.*,αCD and HPαCD were present. In this case, the optimal balance of binding affinity to nonyl chains and hydrophilicity was found with native αCD.

### 2.3. Highly Hydrophobic Copolymers: Poly(2-ethyl-2-oxazoline)-ran-poly(2-nonyl-2-oxazoline) Containing 25% and 30% Nonyl Chains

In Section 2.2 we have seen that a 20% content of NonOx in the copolymer began to induce its self-assembly behavior in solution giving rise to kinetically-trapped nanoparticles. To evaluate the possibility to enhance this behavior, two copolymers with the same chain length (≈100 repeating units) and higher NonOx contents of 25% and 33% were synthesized (SN25 and SN33, respectively). The copolymers were dissolved in the presence of αCD following the same protocol as described for the previous copolymer with 20% NonOx. Due to the higher hydrophobicity of the newly synthesized copolymers, a higher concentration of αCD was necessary to bring them in solution. One equivalent of αCD was required to solubilize SN25, whereas the more hydrophobic SN33 could only be solubilized in a near-saturation concentration of the cavitand. Therefore, the more hydrophilic HPαCD, with a solubility of over 600 mg·mL^−1^ was utilized, whereby more than 1.7 equivalents of the cavitand were necessary to solubilize the copolymer. Strong hydrophobic interactions established among the nonyl chains are proposed to hinder the access of the cavitands to establish host–guest complexes that disrupt the polymer-polymer interactions and progressively extend the polymer chain. Host–guest complex formation is thought to open up new nonyl chains for complexation. Thus, repeated cycles of freezing and thawing the solution under sonication at 0 °C were found to be required to solubilize the copolymer.

**Figure 7:**
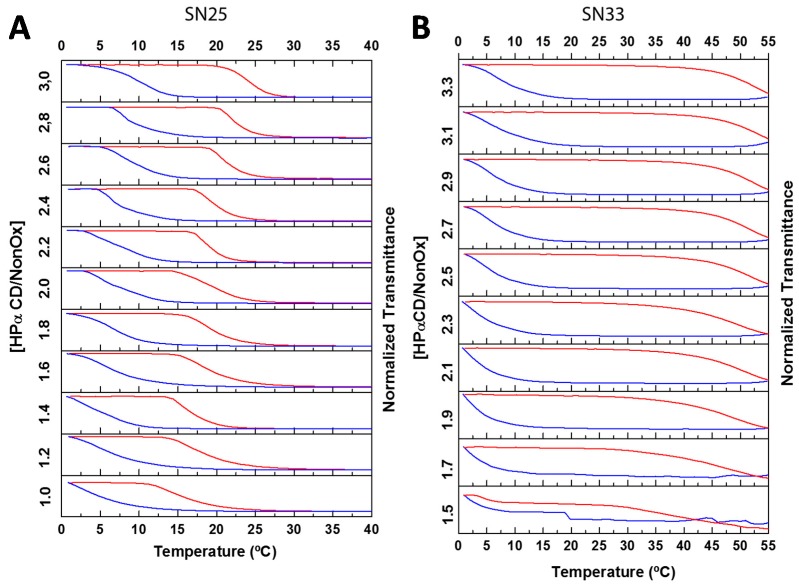
Turbidimetry studies of of 5 mg·mL^−1^ solutions of P[(EtOx)_75_-*ran*-(NonOx)_25_] (**A**, SN25) and P[(EtOx)_62_-*ran*-(NonOx)_29_] (**B**, SN33) with increasing HPαCD/NonOx molar ratios. Both copolymers exhibited minor variations of the T_CP_ upon addition of increasing amounts of cavitand, which is ascribed to nanoparticle formation where the nonyl chains are isolated from the aqueous environment. In the case of SN33, an unprecedented large hysteresis of 40 °C was found. The heating and cooling ramps are represented by the top (red) and bottom (blue) curves, respectively. Rate: 1 K·min^−1^, λ = 700 nm.

Figure 7 shows the results of the titration of both copolymers with HPαCD. In both cases, addition of more cavitand to the solution had a minor effect on the T_CP_. This behavior is ascribed to the formation of kinetically-trapped nanoparticles that contain most of the nonyl chains in the core, isolated from the solution and inaccessible for further host–guest complexation. In the case of SN25, the formation of similar kinetically-trapped nanoparticles as seen for SN20 would explain its homologous thermoresponsive behavior in the presence of HPαCD.

In analogy with SN20 and SN25, increasing the concentration of HPαCD had only a minor effect over the T_CP_ of SN33. However, this copolymer exhibited a hysteresis of unprecedented magnitude (*ca.* 40 K). Once the copolymer-HPαCD ensemble was dissolved at low temperatures, the solution remained transparent up to *ca.* 50 °C, when the solution became white opaque due to aggregation of copolymer-HPαCD nanoparticles. The solution then remained opaque in the whole temperature range, and it was necessary to lower the temperature to *ca.* 0 °C to break the aggregates and recover a transparent solution containing individual nanoparticles. The copolymer-HPαCD ensemble was thus able to remember the thermal history of the solution, resulting in a temperature sensor with memory function. An in-depth study was performed to understand the reasons behind the highly unusual solution behavior of this copolymer-cavitand ensemble and has been reported elsewhere [43]. It is thus apparent that increasing the content of hydrophobic nonyl chains in the copolymer further increases the stability of the kinetically-trapped nanoparticles. These nanoparticles only expose a minor fraction of nonyl chains to the surrounding medium, directing most nonyl chains into the hydrophobic core of the nanoparticle.

### 2.4. Poly(2-ethyl-2-oxazoline)-ran-poly(2-nonyl-2-oxazoline): Influence of Polymer Length

Finally, the effect of polymer chain length on the solubility behavior of the PEtOx-*ran*-PNonOx copolymers was investigated. To this end, two copolymers were synthesized bearing *ca.* 200 repeating units, thus double the length of the copolymers studied previously. The copolymers had a NonOx content of 19% (LN19) or 29% (LN29) and were, as expected, insoluble in the absence of cavitands. In fact, the increase in polymer chain length rendered, as expected, both copolymers more difficult to dissolve, requiring 2 and 4 equivalents of HPαCD to solubilize LN19 and LN29, respectively.

**Figure 8:**
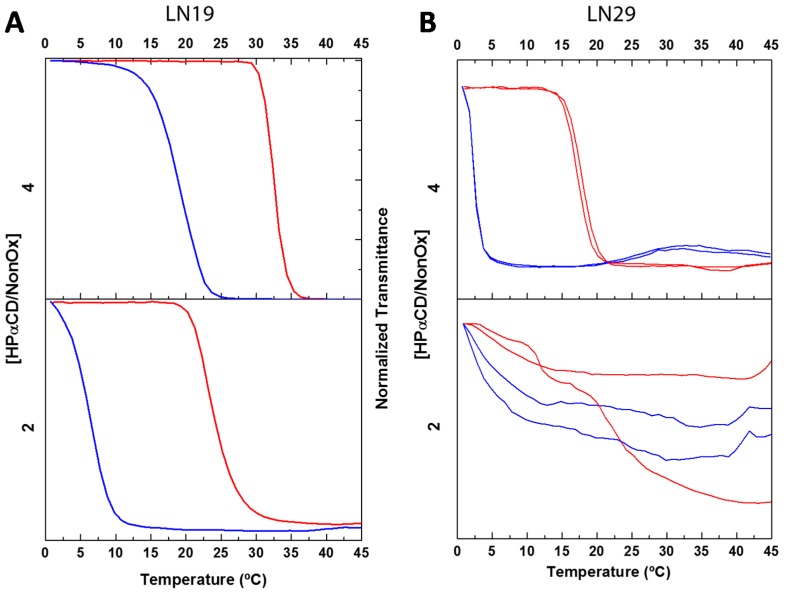
Turbidimetry studies of 5 mg·mL^−1^ solutions of P[(EtOx)_162_-*ran*-(NonOx)_38_] (**A**, LN19) and P[(EtOx)_140_-*ran*-(NonOx)_57_] (**B**, LN29) with increasing HPαCD/NonOx molar ratios. Large HPαCD excesses are required to solubilize both copolymers, especially in the case of LN29. The heating and cooling ramps are represented by the top (red) and bottom (blue) curves, respectively. Rate: 1 K·min^−1^, λ = 700 nm.

Figure 8 shows the temperature dependent turbidimetry results, which yielded a sharp LCST transition in both copolymers, in contrast with the more progressive transition found in the shorter copolymers in the presence of HPαCD. The T_CP_ of LN19 only increased by *ca.* 5 K when increasing the amount of the cavitand from 2 to 4 equivalents, therefore indicating the unavailability of most of the copolymer nonyl chains to engage in host–guest complexation. The higher number of nonyl groups present in a single polymer chain thus seems to enhance the intramolecular nonyl–nonyl hydrophobic interactions further stabilizing the insoluble globular morphology of the copolymer. This results in the need to add a large excess of HPαCD to disrupt the nonyl–nonyl hydrophobic interactions and is most evident in the case of LN29, which requires up to 4 equivalents of HPαCD to be solubilized.

## 3. Experimental Section

### 3.1. Materials

Solvents and reagents were purchased from Sigma Aldrich (St. Louis, MO, USA), and used as received unless otherwise specified. Cucurbit[7]uril was synthesized as reported [49,50]. Methyl tosylate (MeOTs) was distilled twice under vacuum prior to use. 2-Ethyl-2-oxazoline (EtOx), 2-nonyl-2-oxazoline (NonOx, Henkel, Dusseldorf, Germany), were distilled over barium oxide (BaO). Acetonitrile (CH_3_CN, Acros Organics, Geel, Belgium) was dried over molecular sieves (3 Å). All reagents were stored and handled under a dry argon or nitrogen atmosphere.

Deionized (Milli-Q) water was obtained from a Sartorius Arium 611 with a Sartopore 2 150 (0.45 + 0.2 µm pore size) cartridge filter (resistivity ≥18.2 MΩ·cm) (Sartorius, Göttingen, Germany).

### 3.2. Instrumentation

The polymerization was performed in a Biotage initiator sixty microwave synthesizer (Biotage, Uppsala, Sweden) utilizing capped microwave vials. The vials were heated to 120 °C for 24 h and cooled down to room temperature under vacuum prior to use. The polymerization was performed with temperature control (IR sensor)

^1^H-NMR spectroscopy was performed in CDCl_3_ on a Bruker Avance 300 MHz spectrometer (Bruker Corporation, Billerica, MA, USA). Spectra were processed using TOPSPIN 3.0 (Bruker Corporation, Billerica, MA, USA).

Size exclusion chromatography (SEC) measurements were performed on an Agilent 1260-series equipped with a 1260 ISO-pump, a 1260 Diode Array Detector (DAD), a 1260 Refractive Index Detector (RID), and two Mixed-D columns and a Mixed-D guard column (Agilent Technologies, Santa Clara, CA, USA) in series inside a 1260 Thermostated Column Compartment (TCC) at 50 °C using dimethylacetamide containing 50 mM of LiCl (flow rate of 0.6 mL·min^−1^) as solvent. Molar mass and dispersity were calculated against poly(methyl methacrylate) standards.

### 3.3. Turbidimetry and Dynamic Light Scattering Studies

Turbidimetry measurements were performed in a CARY Bio 100 UV–VIS spectrophotometer equipped (Agilent Technologies, Santa Clara, CA, USA) with a temperature controller, at a wavelength of 700 nm. Heating/cooling cycles were performed at a rate of 1 K·min^−1^ with stirring. The polymer concentration was kept at 5 mg·mL^−1^ in deionized water. The equivalents of cyclodextrin and cucurbit[7]uril added were calculated in relation to the equivalents of nonyl side chains.

### 3.4. Poly[(2-ethyl-2-oxazoline)-ran-(2-nonyl-2-oxazoline)] Synthesis

The polymerizations were performed as previously reported [26]. The microwave vials were loaded in a glove box (Vigor Gas Purification Technologies Inc., Houston, TX, USA) with EtOx and NonOx copolymers in the desired molar ratio, maintaining a total monomer concentration of 4 M in acetonitrile. Polymer length was tuned by the ratio of MeOTs initiator to monomer. Table 2 summarizes the amounts of initiator, monomers, and solvent that were used to produce each copolymer.

The polymerizations were run for 15 min at 140 °C (DP 100) or 30 min (DP 200). The polymers were terminated with KOH in methanol, yielding hydroxyl-terminated polymers. The solvent was evaporated under reduced pressure, and each polymer was subsequently precipitated in diethyl ether from dichloromethane. The pure polymer was then dried in a vacuum oven at 50 °C for 24 h.

**Table 2:** Reagents quantities and obtained copolymer compositions.

ID	Target Polymer Composition	Obtained Polymer Composition ^(a)^	DP (Target/Obtained)	MeOTs (g)	EtOx (g)	NonOx (g)	MeCN (mL)
SN12	PEtOx_90_-*ran*-PNonOx_10_	PEtOx_90_-*ran*-PNonOx_12_	100/102	0.0335	1.606	0.355	2.491
SN20	PEtOx_80_-*ran*-PNonOx_20_	PEtOx_84_-*ran*-PNonOx_21_	100/105	0.0272	1.454	0.747	2.299
SN25	PEtOx_75_-*ran*-PNonOx_25_	PEtOx_75_-*ran*-PNonOx_25_	100/100	0.0223	0.892	0.592	1.155
SN33	PEtOx_70_-*ran*-PNonOx_30_	PEtOx_62_-*ran*-PNonOx_29_	100/91	0.0272	1.272	1.120	2.108
LN19	PEtOx_160_-*ran*-PNonOx_40_	PEtOx_162_-*ran*-PNonOx_38_	200/200	0.0109	0.933	0.464	1.502
LN29	PEtOx_140_-*ran*-PNonOx_60_	PEtOx_140_-*ran*-PNonOx_57_	200/197	0.0112	0.833	0.710	1.104

^a^ Calculated by ^1^H-NMR spectroscopy.

### 3.5. Preparation of Poly[(2-ethyl-2-oxazoline)-ran-(2-nonyl-2-oxazoline)] Solutions and Titration

For all the titrations with a cavitand stock solution, PMMA cuvettes for Vis-spectroscopy (Carl–Roth), each equipped with a stirring bar, were filled with 2 mL of copolymer solution. To calculate the amount of cavitand stock solution necessary to add to each cuvette, the following calculations were performed.

First, the weight fraction of NonOx (*f*_NonOx_) in the copolymer was calculated, according to the following equation:

(1) $$\text{Wt}.\text{ fraction NonOx }\left(f_{\text{NonOx}}\right)\;=\frac{DP\;\left(\text{NonOx}\right)M_{wt}\left(\text{NonOx}\right)}{DP\;\left(\text{EtOx}\right)M_{wt}\left(\text{EtOx}\right)+DP\;\left(N\text{onOx}\right)M_{wt}\left(\text{NonOx}\right)}$$

where DP is the degree of polymerization i.e., the number of repeating units of each monomer in the copolymer.

Then, the mass of cavitand necessary to equal the number of NonOx groups contained in 2 mL (cuvette) of 5 mg·mL^−1^ copolymer solution is calculated.

(2) $$m\;cavitand\;\left(\text{grams}\right)=\frac{f_{\text{NonOx}}\;\times \;5{\text{ mg mL}}^{-1}\times \;2\text{ mL}}{M_{wt}\left(\text{NonOx}\right)}\times \;M_{wt}\left(cavitand\right)$$

Finally, the volume of cavitand stock solution required to add to each copolymer solution is calculated:

(3) $$V\;cavitand\;\left(\text{μL}\right)=1000\;\times \;\frac{m\;cavitand\;\left(\text{g}\right)}{concentration\;cavitand\;\left(\frac{\text{g}}{\text{mL}}\right)}\;\times \;Number\;of\;equivalents$$

In the present study, the number of equivalents ranged from 0.2 to 2.0. The aliquots were measured and dispensed with a micropipette.

Since the investigated copolymers were insoluble, a 5 mg·mL^−1^ stock solution was prepared in the presence of cavitand (e.g., 0.2 equivalents HPαCD for SN20, or 1.7 equivalents HPαCD for SN33). This cavitand-copolymer mixture was frozen and thawed under sonication in an ice bath. This protocol was repeated until all macroscopic polymer particles disappeared.

PMMA cuvettes were filled with 2 mL of the copolymer solution. Subsequently, increasing amounts of the cavitand stock solution were added to each cuvette, obtaining the desired cavitand concentration. Cavitand stock solution concentrations ranged from 120 mg·mL^−1^ for αCD to 150 mg·mL^−1^ for HPαCD. The dilution of the copolymer dilution by addition of cavitand was always kept below 10%.

Due to the relatively low solubility of CB7, a 4 mM solution of the cavitand was prepared (4.83 mg·mL^−1^). To avoid high dilution effects upon titration, the copolymer was directly weighted in the cuvette, and the necessary amounts of water and CB7 stock solution were added to obtain a 5 mg·mL^−1^ concentration of copolymer.

## 4. Conclusions

The copolymer composition of PEtOx-*ran*-PNonOx copolymers was found to exert a tremendous impact on its host–guest complexation capabilities and on the structures formed at the nanoscale upon complexation with supramolecular hosts. In our previous study comprising a PEtOx-*ran*-PNonOx copolymer containing 12% NonOx, cavitand-nonyl complexation resulted in the extension of the polymer chain and the formation of random-coil polymer-cavitand ensembles. Increasing the nonyl content to 20%–25% promoted the formation of kinetically-trapped nanoparticles, in which the core nonyl chains are shielded from the aqueous environment. Macroscopically, this translated into a progressive temperature-induced phase transition. When the NonOx content was further increased to 33% kinetically-trapped nanoparticle formation promoted the appearance of a large hysteresis, which resulted in temperature sensors with the potential to exhibit a memory function [43].

Finally, increasing the copolymer chain length to 200 repeating units seemed to favor intramolecular nonyl–nonyl hydrophobic interactions, promoting a polymer globular structure and hindering host–guest complex formation. Thus, large excess of cavitands was required to solubilize these longer copolymers.

Overall, the results obtained demonstrate that, when coupled to a polymer structure, simple and relatively weak supramolecular interactions in water can trigger the formation of a variety of complex, defined architectures at the nanoscale. These supramolecular architectures are determined by subtle changes in molecular structure and dictate the macroscopic behavior of the solution. The larger number of hydrophobic units in water insoluble PEtOx-*ran*-PNonOx copolymers in combination with highly hydrophilic supramolecular hosts, favored the formation of well-defined kinetically-trapped nanoparticles.

Besides advancing in the understanding of the interplay between amphiphilic polymers and supramolecular host molecules, further research could be directed to the use of the obtained nanoparticles in the solubilization of hydrophobic molecules of relevance in biomedical applications.
